# Impact of headache frequency and preventive medication failure on quality of life, functioning, and costs among individuals with migraine across several European countries: need for effective preventive treatment

**DOI:** 10.1186/s10194-023-01655-5

**Published:** 2023-08-24

**Authors:** Dawn C. Buse, Patricia Pozo-Rosich, Laure Dupont-Benjamin, Bridget L. Balkaran, Lulu Lee, Adam Jauregui, Pranav Gandhi, Mousam Parikh, Uwe Reuter

**Affiliations:** 1 Albert Einstein School of Medicine, Bronx, NY USA; 2 Headache Unit, Neurology Department, Vall d’Hebron University Hospital, Barcelona, Spain; 3 Headache Research Group, VHIR, Universitat Autonoma de Barcelona, Barcelona, Spain; 4 AbbVie, Courbevoie, France; 5 Cerner Enviza, Malvern, PA USA; 6 AbbVie, Madison, NJ USA; 7 Charité Universitätsmedizin Berlin, Berlin, Germany; 8 Universitätsmedizin Greifswald, Greifswald, Germany

**Keywords:** Preventive medicine, Migraine, Health-related quality of life, Absenteeism, Presenteeism, Healthcare costs, Treatment failure

## Abstract

**Background:**

Data are limited regarding the combined impact of headache frequency and failure of preventive medication (efficacy and/or tolerability) on the humanistic/economic burden of migraine.

**Methods:**

A retrospective, cross-sectional analysis of 2020 National Health and Wellness Survey (NHWS) data was conducted. An opt-in online survey identified adults in France, Germany, Italy, Spain, and United Kingdom with self-reported physician-diagnosed migraine. Participants with ≥ 4 monthly headache days (MHDs) were stratified by prior preventive medication use/failure (preventive naive; 0–1 failure; ≥ 2 failures). Quality-of-life and economic outcomes were compared among groups using generalized linear modeling.

**Results:**

Among individuals with ≥ 4 MHDs (*n* = 1106), the NHWS identified 298 (27%) with ≥ 2 failures, 308 (28%) with 0–1 failure, and 500 (45%) as preventive naive. Individuals with ≥ 2 failures versus preventive-naive individuals had significantly lower scores on the 12-Item Short Form Survey Physical Component Summary (42.2 vs 44.1; *P* < 0.005), numerically higher scores on the Mental Component Summary (39.5 vs 38.5; *P* = 0.145), significantly higher scores on the Migraine Disability Assessment (39.1 vs 34.0; *P* < 0.05), and significantly higher prevalence of depression symptoms (62% vs 47%; *P* < 0.001) and anxiety symptoms (42% vs 31%; *P* < 0.01). The ≥ 2 failures group versus the preventive-naive group also had significantly more functional impairment as assessed by mean numbers of migraine-specific missed work days (7.8 vs 4.3) and household activities days (14.3 vs 10.6) in the past 6 months (*P* < 0.001) as well as the prevalence of absenteeism (19% vs 13%), overall work impairment (53% vs 42%), and activity impairment (53% vs 47%) (all *P* < 0.05). Emergency department visits (0.7 vs 0.5; *P* = 0.001) and hospitalizations (0.5 vs 0.3; *P* < 0.001) in the past 6 months were significantly higher in the ≥ 2 failures group versus the preventive-naive group, while indirect costs (€13,720 vs €11,282) and the proportion of individuals with non-adherence during the past 7 days (73% vs 64%) were numerically higher.

**Conclusions:**

Increased burden, quality-of-life impairment, and functional impairment exist among individuals with migraine experiencing ≥ 4 MHDs and more treatment failures. While cause and directionality cannot be determined, these results suggest the need for effective preventive migraine treatments.

**Graphical Abstract:**

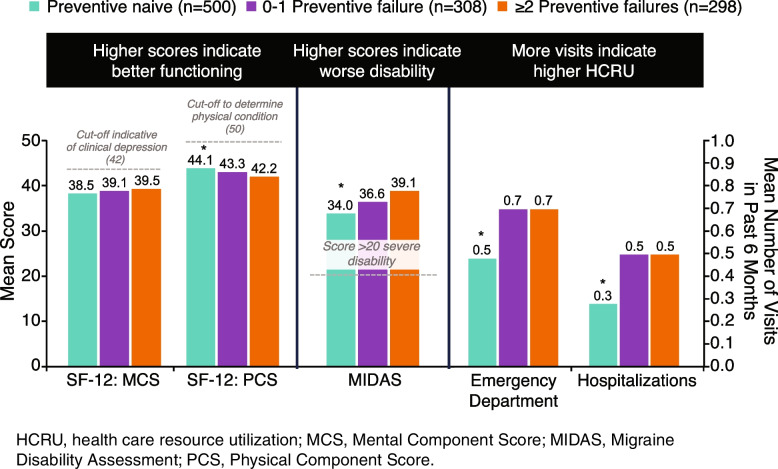

**Supplementary Information:**

The online version contains supplementary material available at 10.1186/s10194-023-01655-5.

## Introduction

Migraine is a debilitating neurological disease that affects more than 1 billion people worldwide [1, 2]. Migraine can be divided into episodic (< 15 monthly headache days [MHDs]) or chronic (≥ 15 MHDs for ≥ 3 months, ≥ 8 of which are migraine days) [2]. It has been demonstrated that as the frequency of daily headaches increases, the burden of migraine consequently increases with each additional headache day, and even those with relatively fewer headache days experience significant negative effects on functioning [3–6]. However, inconsistent definitions have been used to categorize patients by headache frequency for evaluating real-world effects in this population. Defining headache frequency cut scores where a noticeable shift in disability occurs could be useful for clinical and research purposes [3–6]. Among individuals with migraine, approximately 88–93% have episodic migraine and approximately 7–12% have chronic migraine, and an estimated 14–26% report experiencing more than 4 headaches per month [7–10].

Migraine exerts a substantial burden and is the second-highest cause of disability worldwide [11–14]. The burden among people with episodic migraine can be significant; a study by Torres-Ferrús et al. showed that high-frequency episodic migraine and chronic migraine had similar burdens and impacts [15]. Analyses of data from the American Migraine Prevalence and Prevention (AMPP) Study and the American Registry for Migraine Research (both conducted in US populations) as well as the Chronic Migraine Epidemiology and Outcomes (CaMEO) US and International studies also showed similar high burdens among people with high frequency migraine and chronic migraine on a broad range of variables, including disability/impact, comorbidities, and sociodemographics [5, 6, 16]. Data from the CaMEO study showed that the burden and impact of living with migraine increased with increasing headache frequency and was substantial at 4 or more monthly headache days on average in work, school, financial, family, social, and leisure domains [4, 17]. A study by Domane et al. showed that individuals with migraine and ≥ 4 monthly headache days (MHDs) (including individuals with 4–7, 8–14, or ≥ 15 MHDs) have significantly worse health-related quality of life (HRQoL), evaluated using the EuroQol 5-Dimension Health Questionnaire (EQ-5D), compared with those with 1–3 MHDs [7]. Other studies have shown significantly greater disability due to headache, evaluated using the Migraine Disability Assessment Scale (MIDAS), in individuals with high-frequency episodic migraine or chronic migraine compared with individuals with low/moderate-frequency episodic migraine [4–6]. In addition, individuals with 8–14 or ≥ 15 MHDs reported significantly higher absenteeism, presenteeism, total work productivity impairment, and activity impairment compared with those with 1–3 MHDs [7]. Health care resource utilization (HCRU), as measured by mean number of health care provider and neurologist visits, was higher among individuals with migraine and ≥ 4 MHDs compared with those with migraine and 1–3 MHDs [7].

Preventive medication can fail for patients based on lack of efficacy, lack of tolerability, other reasons such as access, or a combination of factors. In a 31-country study of 11,266 individuals with migraine and ≥ 4 MHDs, Martelletti et al. found that those who experienced ≥ 2 preventive treatment failures reported a significantly higher impact of migraine on their professional lives, compared with those who had not experienced preventive treatment failure [18]. In another study, Ford et al. evaluated the burden of migraine among patients cycling through migraine preventive treatments and demonstrated that individuals who had cycled through multiple medication classes had more health care visits, emergency department visits, inpatient admissions, and acute medication use [19]. All-cause and migraine-related total costs were significantly higher among individuals with migraine who discontinued their first or second preventive medication class compared with those who were persistent with their initial preventive medication class [19]. While it is known that the burden of headache differs by frequency or preventive treatment failure individually [18–20], to our knowledge the Burden of migrainE in specialist headache Centers treating patients with prOphylactic treatMent failure (BECOME) study is the only prior analysis to have assessed the combined effect of headache frequency and prior preventive medication use and failure among individuals with migraine [21].

The objective of this study was to evaluate unmet needs among persons with migraine in France, Germany, Italy, Spain, and the United Kingdom (UK), focusing specifically on the role of self-reported prior preventive medication failure and headache frequency in the humanistic and economic burdens of migraine. Individuals with a self-reported physician diagnosis of migraine who differed by preventive medication failure history were compared in terms of demographics, health characteristics, treatment characteristics, and health outcomes.

## Methods

### Study design

This study was a non-interventional, retrospective, cross-sectional analysis of data collected in 2020 as part of the National Health and Wellness Survey (NHWS) from respondents located in France, Germany, Italy, Spain, and the UK. The 2020 NHWS data were reviewed and granted exemption status by the Pearl Institutional Review Board (Indianapolis, IN).

### Data source

The NHWS is a self-administered, internet-based survey that is administered to adults in several countries. Respondents were required to be ≥ 18 years of age, able to read and write the primary language of the country in which the study was conducted (French, German, Italian, Spanish, or English), and to provide informed consent. The survey consists of a base questionnaire that assesses demographics and health characteristics, and disease-state specific modules that are only completed by respondents who self-report a diagnosis of the disease state.

Potential respondents were identified primarily through participation in opt-in online survey panels, with stratified quota sampling to ensure country-specific representation in terms of age and gender. Domestic and international data from the Current Population Survey of the US Census were used to identify the relative proportions of age and gender in each country surveyed. These demographic data from different countries are collected by the US Census Bureau from international sources and were used for the current study because the US Census Bureau is a reputable source. These proportions were then replicated with the recruitment of panel members using a stratified quota sampling framework to ensure that the final NHWS sample matched the demographic distribution of each country surveyed. To further ensure a representative sample, particularly among those > 65 years of age in Spain and Italy, where internet penetration was considered insufficient to provide an adequate sample, online panel recruitment was supplemented by computer-assisted web interviews in which respondents were recruited via telephone and could choose to complete the interview on the phone, on a computer in a private center, or through an emailed link.

### Study population

In this analysis, respondents who were residents of France, Germany, Italy, Spain, or the UK, ≥ 18 years of age, agreed to informed consent, had a self-reported physician diagnosis of migraine, and had ≥ 4 MHDs were evaluated. Study cohorts were stratified by prior preventive medication failure history. Preventive medication failure was stratified as “preventive treatment naive,” “0–1 preventive failures,” and “ ≥ 2 preventive failures.” Preventive failure was defined as self-reported non-response to treatment. A preventive medication failure history of 0 referred to respondents who currently or previously took at least 1 preventive medication for headache and had not reported failure.

### Outcomes

#### Demographics and health characteristics

Demographic and general health variables were evaluated, including age, gender, marital status, education, household income, insurance type, body mass index, smoking status, alcohol use, and exercise behavior. The Charlson Comorbidity Index (CCI) [22] was also assessed. The CCI represents a weighted sum of multiple comorbid conditions predictive of mortality, with higher CCI scores indicating a greater comorbid burden for the patient.

In addition, several migraine-specific variables were assessed, including time since diagnosis, migraine severity (self-rated as mild, moderate, or severe when using and not using medication), migraine diagnostician (i.e., primary care provider, nurse practitioner, neurologist, etc.), and migraine-specific preventive and acute medication usage and over-the-counter (OTC) medication use. Respondents were initially asked which medications they were using and whether they were used for treating or preventing migraine. Those who described their medications as being used for treatment were included in the “acute medication use” cohort, while OTC treatments were defined as non-prescription migraine medications. The number of days that these treatments were used in the past month and the number typically used per month to treat a migraine attack were also evaluated. Mean number of headache days in the past 30 days and number of prior preventive migraine medications failed were collected to inform how cohorts were composed.

### Health-Related Quality of Life (HRQoL)

Analyses evaluated patient-reported HRQoL using responses to validated instruments, including the 12-Item Short Form Survey Instrument (SF-12) [23, 24], the EQ-5D-5L [25], the MIDAS [26], the Patient Health Questionnaire (PHQ-9) [27], and the Generalized Anxiety Disorder scale (GAD-7) [28].

### 12-Item Short Form Survey Instrument (SF-12)

The SF-12 is a 12-item, multipurpose, HRQoL instrument that is a generic measure of health status and uses norm-based scoring to allow for comparisons with other generic health surveys. Two summary scores of the SF-12 were calculated: physical component summary (PCS) and mental component summary (MCS). In this analysis, SF-12 PCS and MCS scores were utilized as normed scores, which was achieved by transforming the raw scores for the items to a mean of 50 and a standard deviation (SD) of 10. Scores can be interpreted relative to a population average of 50 and with other comparison groups of interest. Higher SF-12 PCS and MCS scores indicate better physical and mental health functioning.

The SF-12 can also be used to generate a health state utilities index. This score is achieved through the application of the SF-6D algorithm, which incorporates 6 domains from the SF-12. The SF-6D utilities index is a preference-based single-index measure for health using general population values. The SF-6D index has interval scoring properties and yields summary scores on a theoretical 0–1 scale, with higher SF-6D index scores indicating a better health state. Analyses for this study included SF-12 PCS, SF-12 MCS, and SF-6D scores [23, 24].

### EQ-5D-5L

The EQ-5D-5L is a self-report measure of health for clinical and economic appraisal. It consists of a descriptive system and a visual analog scale (VAS). The EQ-5D descriptive system includes 5 dimensions: mobility, self-care, usual activities, pain/discomfort, and anxiety/depression. Respondents score each dimension using 5 levels. Scores on these dimensions are combined to create a summary index score, the EQ-5D Utility Index, with higher EQ-5D Index scores representing a better health state. The EQ-VAS asks respondents to linearly score their self-rated health with the endpoints of the line being “best imaginable health state” and “worst imaginable health state.” Analyses for this study included the EQ-5D Utility Index scores and the EQ-VAS scale [25].

### Migraine Disability Assessment Scale (MIDAS)

The MIDAS evaluates the impact of headaches on daily activities and provides a measure of “functional disability” [26]. The 7-item instrument assesses work/school absences and presenteeism, household productivity loss, and impact on social and family activities. Scores are summed, with higher scores representing greater levels of functional disability. The instrument also captures individual differences in usual headache pain intensity on a VAS of 0–10. Analyses for this study included the MIDAS sum score from the first 5 items and the VAS pain score. MIDAS scores were interpreted using the standard grading system for the MIDAS questionnaire (score 0–5, none, minimal or infrequent disability; score 6–10, mild or infrequent disability; score 11–20, moderate disability; score > 20, severe disability) [26].

### Work and activity impairment

Work productivity was assessed using the Work Productivity and Activity Impairment General Health (WPAI-GH) questionnaire, a 6-item validated instrument with 4 metrics: absenteeism (the percentage of work time missed because of one’s health in the past 7 days), presenteeism (the percentage of impairment experienced while at work in the past 7 days because of one’s health), overall work productivity loss (an overall impairment estimate that is a combination of absenteeism and presenteeism), and activity impairment (the percentage of impairment in daily activities because of one’s health in the past 7 days). Only respondents who reported being full-time, part-time, or self-employed provided data for absenteeism, presenteeism, and overall work impairment. All respondents provided data for activity impairment.

The WPAI is part of the general module completed by all NHWS respondents and is not part of the migraine-specific module of the NHWS. Thus, the general health version used in this study is the best option available for this population because no migraine-specific version is available. Migraine-specific work days missed and migraine-specific household activities days missed were also assessed with 2 custom survey items that were designed to follow the MIDAS questionnaire (see Additional file 1: Supplemental Table 1).

### Depression and anxiety symptoms

The PHQ-9 is used to screen for depression, and provides a measure of depression symptom severity, using a sum scoring of items representing diagnostic criteria from the *Diagnostic and Statistical Manual of Mental Disorders (DSM)-IV* [27, 29]. There is also an algorithm scoring method that more closely approximates the *DSM-IV* clinical scoring algorithm, which assesses 2 cardinal depressive symptoms first; however, we used the sum scoring method. The PHQ-9 measures frequency of depression symptoms using items scored on a 4-point scale. A tenth item on the PHQ-9 is not scored and assesses the impact of depression-related problems on a respondent’s ability to function. The total sum score ranges from 0 to 27. A score of 10–14 indicates moderate depression, 15–19 indicates moderate-to-severe depression, and 20–27 indicates severe depression. Depression severity was examined in analyses for this study, and PHQ-9 scores were bucketed, as is standard practice.

The GAD-7 assesses the severity of anxiety symptoms using a 7-item general anxiety measure with a format similar to that of the PHQ-9 [28]. Individuals rate how bothered they have been by different *DSM-IV* generalized anxiety criteria symptoms over the prior 2 weeks, with each item scored from 0 to 3, providing a 0–21 severity score for anxiety symptomology. Scores of 5, 10, and 15 represent cutoff scores for mild, moderate, and severe anxiety symptoms, respectively. A single item for how difficult the symptoms have made it to do work, take care of the home, or get along with others is also included. Anxiety severity was examined in analyses for this study and GAD-7 scores were compiled according to standard practice.

### Functional impairment and HCRU

The economic burden of migraine was evaluated using patient-reported responses to the validated WPAI-GH questionnaire [30] and Medications Adherence Reasons (MAR) Scale (described below) [31]. Indirect costs associated with lost work productivity were estimated for each respondent using a human capital method. Patients also were asked to report HCRU in the past 6 months.

### Indirect costs

Indirect costs were estimated from NHWS data following established procedures that have been published previously [32, 33]. Indirect costs associated with lost work productivity were estimated for each respondent using the human capital method. Indirect costs were calculated by integrating data from Eurostat median hourly wages by country and gender for 2014 [34, 35], and then inflated to 2020 values using data on projected wage growth obtained from Wage Developments in the Euro Area [35]. The number of hours respondents missed due to absenteeism and the number of hours they missed due to presenteeism were each multiplied by their associated hourly wage. These figures (which represent societal lost earnings per employee per week for absenteeism and presenteeism, respectively) were annualized by multiplying by the country’s average work weeks per year to obtain total annual estimates [36]. The annual costs for absenteeism and presenteeism were combined to calculate total indirect costs.

### HCRU

HCRU was defined as the number of all-cause visits in the past 6 months with a traditional health care provider (HCP) (eg, pulmonologist, gynecologist, psychologist, psychiatrist), general practitioner (GP), internist, and/or neurologist and the number of emergency department visits and hospitalizations.

### Adherence (MAR Scale)

The MAR Scale assesses adherence and a range of reasons for non-adherence [31]. The scale was developed based on literature reviews and patient interviews. The version of the scale used in this study consisted of 20 questions: 1 global adherence question (number of days in the past week that medicine was taken exactly as prescribed) and 19 questions on specific reasons for medication non-adherence (yes/no). The reasons for non-adherence encompass the domains of: (1) logistic issues, or challenges in getting access to the medicine; (2) belief issues, such as low perceived need for the medicine; (3) forgetfulness issues; and (4) long-term concerns (eg, side effects).

In the NHWS, these 20 questions are asked for each applicable mode of administration (ie, oral, self-injection, topical). If multiple medications are taken via the same route, the MAR Scale questions are only asked once for each route. For the purposes of this study, 1 adherence variable and 19 reasons for non-adherence variables were calculated for each route of administration taken by each respondent. Scores for each route of administration were assessed.

### Statistical methods

Multivariable models were used to compare differences in outcomes between prior preventive failure groups, with the ≥ 2 preventive treatment failure population serving as the reference group, while controlling for confounding differences in baseline characteristics. Covariates included in the models were age, sex, insurance status, body mass index categories, smoking history, alcohol intake, exercise, CCI, PHQ-9 score, and GAD-7 score, with the latter 2 covariates excluded when used as outcomes. Adjustment for depression and anxiety could potentially account for unexpected results in SF-12 MCS scores. Prior research has shown that depression and anxiety scores have accounted for a large proportion of the variance in SF-36 MCS scores [37]. Therefore, depression and anxiety were removed from the SF-12 MCS and PCS models while keeping all other original covariates.

Generalized linear models (GLMs) and normal distributions with identity link functions were used for predicting normally distributed outcomes. If normality was not observed for a continuous outcome variable, a GLM approach specifying the appropriate distribution (Gaussian, Poisson, binominal, or negative binomial) was conducted. Logistic models were used for predicting dichotomous outcomes. Regression coefficients, adjusted means, standard errors, rate ratios, *P* values, and confidence intervals were calculated.

As this was on opt-in online survey, only respondents who provided data were included. The NHWS survey does not allow for missing responses and data were only “missing” in a sense due to survey skip logic (ie, respondents who did not qualify to answer a question were not asked to answer that question). Therefore, there was no methodology used to account for missing data.

## Results

From the NHWS data, a total of 7311 patients self-reported having received a diagnosis of migraine from a physician in France, Germany, Italy, Spain, or the UK (mean age 43 years; female 71%; see Additional file 1: Supplemental Table 2). Of these, 1143 (16%) were identified as having ≥ 4 MHDs and included in the full migraine cohort and 1106 (15%) were included in the multivariate analysis population (298/1106 [27%] with ≥ 2 failures, 308/1106 [28%] with 0–1 failure, and 500/1106 [45%] as preventive naive). Based on the full migraine cohort (n = 1143), individuals with ≥ 2 failures at baseline were more likely to be younger and have a higher CCI score compared with those who were preventive naive or had 0–1 failures (Table 1). Individuals with ≥ 2 failures were more likely to have a shorter time since diagnosis and be treated by a neurologist and less likely have acute medication usage compared with those who were preventive-naive or had 0–1 failure. The mean number of OTC medications that individuals reported using to treat migraine in a month (the survey allowed a maximum of 3) was also higher in the ≥ 2 failures group compared with the preventive-naive and 0–1 failure groups. However, days of acute medication use were highest in the preventive-naive group. Individuals with ≥ 2 failures were more likely to be currently exercising and less likely to use alcohol compared with those who were preventive naive or had 0–1 failures.

**Table 1:** Baseline characteristics

**Characteristic**	**Preventive Naive (*n* = 516)**	**0–1 Preventive Failures (*n* = 320)**	≥ **2 Preventive Failures (*n* = 307)**	***P* value**
**Age, mean (SD), y**	44.0 (14.9)	42.2 (13.9)	39.8 (13.8)	0.0002
**Female, n (%)**	381 (74)	212 (66)	222 (72)	0.0560
**Mean Charlson Comorbidity Index score (SD)**	0.34 (0.80)	0.36 (0.86)	0.58 (1.8)	0.0116
**Married/living with partner, n (%)**	324 (63)	202 (63)	189 (62)	0.8912
**University degree or higher, n (%)**	220 (43)	152 (48)	155 (50)	0.1589
**Household income, n (%)**				0.5015
< €20,000	174 (34)	94 (29)	85 (28)	
€20,000 to < €40,000	176 (34)	113 (35)	110 (36)	
€40,000 +	136 (26)	97 (30)	97 (32)	
**Labor force participation, n (%)**	340 (66)	234 (73)	225 (73)	0.0273
**Public insurance, n (%)**	373 (72)	228 (71)	195 (64)	0.0870
**BMI category,**^**a**^** n (%)**				0.5568
Underweight: < 18.5	31 (6)	15 (5)	25 (9)	
Normal: 18.5 to 24.9	251 (50)	147 (48)	132 (46)	
Overweight: 25.0 to 29.9	133 (27)	91 (30)	81 (28)	
Obese: ≥ 30	85 (17)	55 (18)	51 (18)	
**Current/former smoker, n (%)**	305 (59)	210 (66)	190 (62)	0.1689
**Current alcohol use, n (%)**	351 (68)	244 (76)	205 (67)	0.0148
**Current exercise, n (%)**	321 (62)	221 (69)	218 (71)	0.0182
**Mean time since diagnosis (SD), y**	30.02 (15.17)	28.48 (13.12)	25.06 (13.12)	< 0.0001
**Migraine severity, n (%)**				0.1106
Mild	155 (35)	80 (28)	70 (26)	
Moderate	207 (46)	150 (53)	144 (53)	
Severe	87 (19)	54 (19)	57 (21)	
**Migraine diagnostician, n (%)**				< 0.0001
Primary/general practitioner	342 (68)	176 (57)	137 (46)	
Nurse practitioner/physician assistant	6 (1)	8 (3)	6 (2)	
Neurologist	142 (28)	121 (39)	137 (46)	
**Current preventive use, n (%)**	0	54 (17)	54 (18)	< 0.0001
Oral preventive	0	50 (93)	43 (80)	0.0950
Injectable preventive	0	3 (6)	12 (22)	0.0260
**Acute medication use, n (%)**	420 (81)	188 (59)	213 (69)	< 0.0001
**Mean days of acute migraine-specific medication use in past month (SD)**	10.07 (8.44)	10.16 (8.06)	11.16 (9.38)	0.2969
**Mean count of acute migraine-specific medications (SD)**	8.51 (10.92)	7.48 (10.37)	8.33 (12.78)	0.5809
**Mean days of OTC medication in past month (SD)**	7.55 (7.97)	9.94 (9.22)	8.70 (8.13)	0.0105
**Mean count of OTC medications (SD)**	1.50 (0.74)	1.43 (0.70)	1.65 (0.85)	0.0150

*BMI* Body mass index, *OTC* Over-the-counter, *SD* Standard deviation

^a^Some participants had missing BMI data

### Health-Related Quality of Life (HRQoL)

**Fig. 1:**
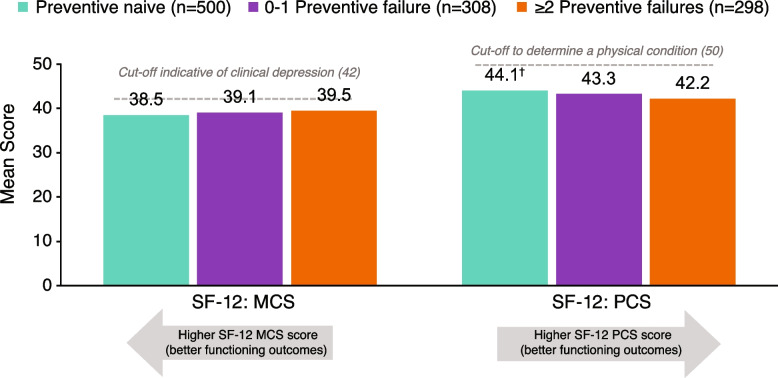
Health-related quality of life: mental health and physical functioning (SF-12 MCS, SF-12 PCS). Results were adjusted for age, sex, country, insurance type, body mass index category, smoking history, alcohol intake, exercise, and CCI. ^†^*P* < 0.01 vs ≥ 2 preventive treatment failures group. CCI, Charlson Comorbidity Index; GAD-7, Generalized Anxiety Disorder-7; MCS, Mental Component Score; PCS: Physical Component Score; PHQ-9, Patient Health Questionnaire-9; SF-12: Short Form-12. Cut-off values from Ware et al. [38]

Mean MCS scores were numerically higher (39.5 vs 38.5; *P* = 0.145) and mean PCS scores were significantly lower (42.2 vs 44.1; *P* < 0.01) in individuals with ≥ 2 failures compared with those who were preventive naive (Fig. 1), indicating improved functioning on the PCS subscale [38]. Results were numerically higher on the MCS scale (39.5 vs 39.1; *P* = 0.627) and numerically lower on the PCS scale (42.3 vs 43.3; *P* = 0.153) in the ≥ 2 failures group compared with the 0–1 failure group, respectively. SF-6D health utilities index scores (0.58 vs 0.58 vs 0.58 in the ≥ 2 failures, 0–1 failure, and preventive-naive groups, respectively) and EQ-5D index scores (0.59 vs 0.60 vs 0.62 in the ≥ 2 failures, 0–1 failure, and preventive-naive groups, respectively) did not differ significantly among groups. EQ-5D VAS scores did not differ significantly among groups (52.0 vs 52.8 vs 52.6 in the ≥ 2 failures, 0–1 failure, and preventive-naive groups, respectively). Although there were no significant differences between groups on EQ-5D scores, there was a trend toward higher HRQoL in the preventive-naive group relative to the ≥ 2 failures group.

**Fig. 2:**
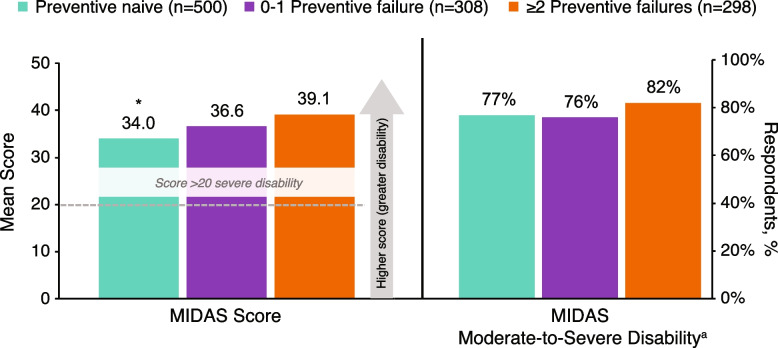
Health-related quality of life: functional disability (MIDAS total score and disability categories). Results were adjusted for age, sex, country, insurance type, body mass index category, smoking history, alcohol intake, exercise, CCI, PHQ-9, and GAD-7. *P < 0.05 vs ≥ 2 preventive treatment failures group. ^a^No/Little or mild disability vs moderate/severe disability. Criterion for disability was MIDAS score ≥ 11. CCI, Charlson Comorbidity Index; GAD-7, Generalized Anxiety Disorder-7; MIDAS, Migraine Disability Assessment; PHQ-9, Patient Health Questionnaire-9

**Fig. 3:**
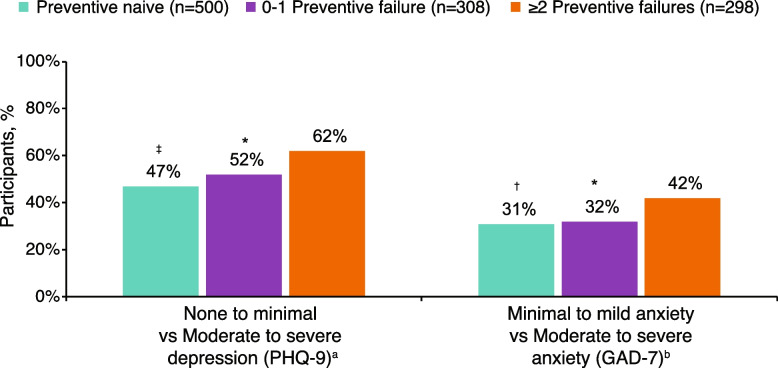
Moderate to severe depression and anxiety symptomology (PHQ-9 and GAD-7). Results were adjusted for age, sex, country, insurance type, body mass index category, smoking history, alcohol intake, exercise, and CCI. *P < 0.05, ^†^*P* < 0.01, ^‡^*P* < 0.001 vs ≥ 2 preventive treatment failures group. ^a^Criterion for “moderate to severe depression” was PHQ-9 score ≥ 10. ^b^Criterion for “moderate to severe anxiety” was GAD-7 score ≥ 10. CCI, Charlson Comorbidity Index; GAD-7, Generalized Anxiety Disorder-7; PHQ-9, Patient Health Questionnaire-9

Mean MIDAS scores were significantly higher in the ≥ 2 failures group compared with the preventive-naive group (39.1 vs 34.0; *P* < 0.05; Fig. 2), indicating more severe disability in the ≥ 2 failures group. Mean MIDAS scores were numerically higher in the ≥ 2 failures group versus the 0–1 failure group (39.1 vs 36.6; *P* = 0.370) but were not statistically significant. MIDAS VAS scores did not differ significantly among groups (7.1 vs 7.2 vs 7.2 in the ≥ 2 failures, 0–1 failure, and preventive-naive groups, respectively). The proportion of participants with moderate to severe migraine-related disability were numerically higher in the ≥ 2 failures group (82%) compared with the preventive-naive (77%) and 0–1 failure (76%) groups. Nonetheless, a significantly higher proportion of patients with moderate to severe depression (62% vs 52% vs 47%, respectively) or anxiety (42% vs 32% vs 31%, respectively) were in the ≥ 2 failures group in relation to the 0–1 failure and preventive-naive groups (*P* < 0.05; Fig. 3).

### Functional Impairment and HCRU

**Fig. 4:**
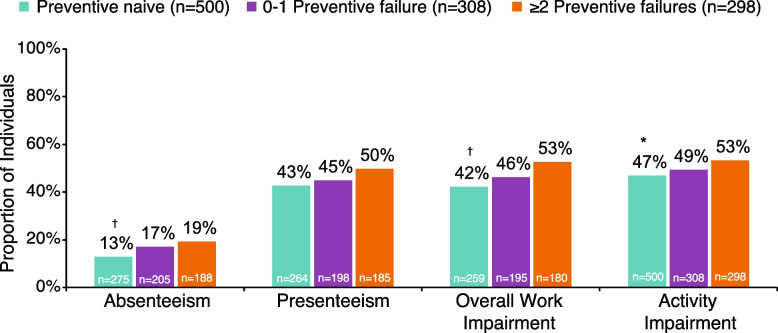
Health-related economic burden: WPAI subscales. Results were adjusted for age, sex, country, insurance type, body mass index category, smoking history, alcohol intake, exercise, CCI, PHQ-9, and GAD-7. *P < 0.05, ^†^*P* < 0.01 vs ≥ 2 preventive treatment failures group. CCI, Charlson Comorbidity Index; GAD-7, Generalized Anxiety Disorder-7; PHQ-9; Patient Health Questionnaire-9; WPAI, Work Productivity and Activity Impairment

The proportions of individuals experiencing absenteeism (19% vs 13%; *P* < 0.01), overall work impairment (53% vs 42%;* P* < 0.01), and activity impairment (53% vs 47%; *P* < 0.05) were significantly higher in the ≥ 2 failures group compared with the preventive-naive group (Fig. 4). Indirect costs were numerically higher in the ≥ 2 failures versus the 0–1 failure and preventive-naive groups (€13,720 vs €11,168 vs €11,282 per year, respectively). Mean number of missed migraine-specific work days (7.8 vs 4.3; *P* < 0.001) and missed migraine-specific household activities days (14.3 vs 10.6; *P* < 0.001) in the past 6 months were significantly higher in the ≥ 2 failures group in relation to the preventive-naive group (Table 2). Mean number of missed migraine-specific household activities days in the past 6 months was significantly higher in the ≥ 2 failures group compared with the 0–1 failures group (14.3 vs 12.0; *P* < 0.05).

**Table 2:** Health-related quality of life: work and household activities days missed due to migraine

Group	Work Days Missed Due to Migraine^a^		Household Activity Days Missed Due to Migraine^a^	
	**Mean (SE)**	***P* value**^**b**^	**Mean (SE)**	***P* value**^**b**^
**Preventive naive (*n* = 500)**	4.3 (0.6)	< 0.001	10.6 (0.8)	< 0.001
**0–1 preventive failures (*n* = 308)**	7.0 (1.0)	0.281	12.0 (1.0)	0.043
≥ **2 preventive failures (*n* = 298)**	7.8 (1.1)	––	14.3 (1.2)	––

Results were adjusted for age, sex, country, insurance type, body mass index category, smoking history, alcohol intake, exercise, CCI, PHQ-9, and GAD-7. Higher numbers of days missed are reflective of poorer outcomes

*CCI* Charlson Comorbidity Index, *GAD-7* Generalized Anxiety Disorder-7, *PHQ-9* Patient Health Questionnaire-9, *SE* Standard error

^a^Days in the past 6 months

^b^*P* value vs ≥ 2 preventive treatment failures group

**Fig. 5:**
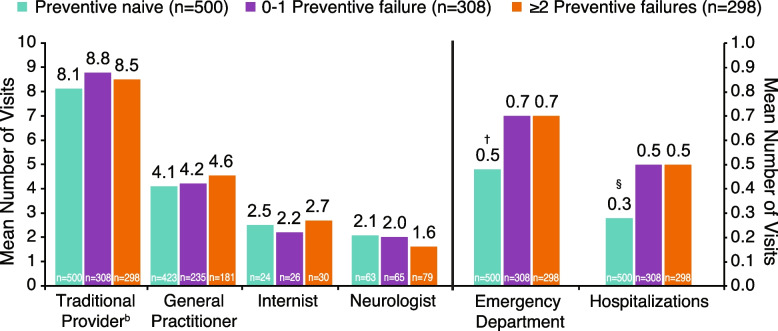
Health-related economic burden: health care resource utilization.^a^ Results were adjusted for age, sex, country, insurance type, body mass index category, smoking history, alcohol intake, exercise, CCI, PHQ-9, and GAD-7. ^†^*P* < 0.001, ^§^*P* = 0.001 vs ≥ 2 preventive treatment failures group. ^a^Visits in the past 6 months. ^b^Traditional providers included pulmonologist, gynecologist, psychologist, etc. CCI, Charlson Comorbidity Index; GAD-7, Generalized Anxiety Disorder-7; PHQ-9; Patient Health Questionnaire-9

Emergency department visits (0.5 vs 0.7;* P* = 0.001) and hospitalizations (0.3 vs 0.5; *P* < 0.001) in the past 6 months were significantly lower in the preventive-naive than in the ≥ 2 failures group (Fig. 5). Adherence to medication rates as assessed by the MAR Scale were 35% (perfectly adherent) and 65% (non-adherent for one or more reasons). The proportion of individuals with non-adherence in the past 7 days was numerically higher in the ≥ 2 failures group compared with the 0–1 failure and preventive-naive groups (73% vs 66% vs 64%, respectively).

## Discussion

This retrospective analysis of nationally representative samples of patient-reported survey (NHWS) data from France, Germany, Italy, Spain, and the UK demonstrated the humanistic and economic burden of migraine among individuals with prior preventive treatment failures. The results from this study demonstrate the significant burden among individuals with migraine and ≥ 2 preventive treatment failures compared with individuals with migraine who had 0–1 preventive treatment failure or were preventive naive, highlighting the need for effective migraine preventive treatment in this population.

This study identified significant differences between individuals who had ≥ 2 preventive failures and those who were preventive naive. The ≥ 2 preventive failures group comprised individuals who were younger, had shorter time since diagnosis, and were more likely to have worse comorbidity scores compared with those with 0–1 failure and those who were preventive naive. Several of these findings are expected, but some are counterintuitive—including the finding that individuals in this group were younger and had a shorter time since diagnosis, as those with ≥ 2 failures are more likely to have a more severe disease profile. In addition, it may seem counterintuitive that individuals with ≥ 2 failures were more likely to be participating in the labor force and currently exercising and were less likely to have current alcohol use compared with those with 0–1 failure and those who were preventive naive. This may be due to the fact that patients who have already failed previous therapies may be more invested in managing their health conditions to control their migraine. The younger age of the ≥ 2 failures patients may have influenced the shorter time to diagnosis and higher percentage of labor force participation. Patients in the preventive-naive group were the most likely to have acute medication usage, which may be due to these patients managing their disease state acutely.

Individuals with ≥ 2 prior preventive treatment failures had significantly worse SF-12 PCS, MIDAS, PHQ-9, and GAD-7 scores and significantly higher numbers of work days and household activity days missed due to migraine compared with those who were preventive naive. Individuals with ≥ 2 prior preventive treatment failures also had significantly more missed household activities days and worse depression and anxiety scores compared with those with 0–1 failure. Rates of absenteeism, overall work and activity impairment, emergency department visits, and hospitalizations were significantly worse for those with ≥ 2 prior preventive treatment failures compared with those who were preventive naive. Individuals with ≥ 2 failures had numerically higher indirect costs and worse adherence to medications during the past 7 days compared with those with 0–1 failure and those who were preventive naive. Thus, individuals with more severe disease are likely to be less adherent to medications and therefore inadequately treated, which may lead to disease progression. These findings demonstrate the high burden of severe migraine among individuals who report lower adherence, perhaps due to polypharmacy or a low belief that a medication will be effective.

Previous studies have demonstrated the burden of migraine by headache frequency [3, 4, 6]. Results from a cross-sectional analysis of 2016 and 2017 NHWS data found that participants with migraine who had ≥ 4 MHDs had greater incremental burden on HRQoL, work and activity impairment, and HCRU compared with those without migraine and those with 1–3 MHDs [7, 20]. Additionally, reductions in MHDs have been associated with improved HRQoL, work and activity impairment, and HCRU [39]. In the AMPP study, participants with high-frequency episodic migraine or chronic migraine were found to have significantly higher rates of severe headache-related disability than those with low-frequency episodic migraine [5]. The Spanish Atlas study demonstrated that higher MHDs were associated with increased migraine-related disability and costs and lower HRQoL [40]. Similarly, results from the BECOME study demonstrated worsening HRQoL and increasing societal burden among patients with migraine and higher MHDs [21]. An analysis of data from the CaMEO study highlighted the burden of migraine on wide-ranging aspects of participants’ lives, including marital and romantic relationships, parenting, career achievement, and financial stability, with greater burdens consistently reported among participants with more MHDs [4]. Recently, an analysis of a cross-sectional online survey (Migraine in Poland study) demonstrated that individuals experiencing migraine without aura had a substantial burden of disease based on MIDAS scores. Severe impairment in performing work or everyday activities was observed in 18% of the participants, including high rates of absenteeism and presenteeism [41].

Previous work has also shown that patients with more treatment failures experience increased migraine-related burden [21]. In the BECOME study, patients who had ≥ 4 prior preventive treatment failures reported more significant impact of migraine (evaluated using Headache Impact Test-6) and more severe disability (evaluated using MIDAS scores) compared with those with 1 prior preventive treatment failure regardless of headache frequency [21]. Patients who had a higher number of prior preventive treatment failures also had more neurologist visits, hospitalizations, and emergency department visits compared with those with 1 prior preventive treatment failure [21].

This study further adds to the existing knowledge base and is one of the few studies to demonstrate the burden of migraine solely from the patient perspective in terms of HRQoL (as opposed to MHDs), a criterion that is underrecognized. The survey provided comprehensive data on multiple disease conditions in a general population that is more reflective of the patient population treated in clinical practice, which may provide different insights into the patient experience. As the study evaluated outcomes among individuals with migraine and increasing numbers of prior treatment failures, the findings have implications for clinical practice, as the increased burden in individuals with ≥ 2 prior preventive treatment failures highlights the need to diagnose and treat patients early with optimized pharmacologic and nonpharmacologic regimens.

There are several limitations of this study. First, all survey data collected in this study are self-reported and are, therefore, associated with potential biases, such as inaccurate recall and false reporting. Additionally, it is not possible to independently verify the reported variables via an additional data source (electronic medical records, physician reports, etc.). However, the NHWS survey is naturalistic and does not present any incentive to misrepresent one’s reporting. Second, there are inherent limitations with the representative nature of NHWS data used in this study. Even though the NHWS is a nationally representative general population survey that uses stratified quota sampling to recruit respondents, most respondents are recruited online. Therefore, these data may not account for the representation of certain groups, including those without access to the internet. However, several studies show that disease prevalence estimates and health outcomes results reported from studies using NHWS data align with results from studies that use non-NHWS data [42–46]. Online surveys have become the most frequently used form of collecting qualitative data due to several advantages, including lower costs, reduced implementation time, less frequent recording errors, and more efficient data analysis [47]. There is also the potential that nonprobability online samples can produce population estimates comparable to those from more traditional probability-based survey research and population benchmarks. Third, there may be country-level differences in the dataset that could not be detected in this study. Small sample sizes preclude the possibility of analyzing results at the individual country-level. Fourth, all data collected are cross-sectional in nature. Thus, associations can be measured, but causality cannot be assessed. Confounding by indication should be considered when interpreting these results. Fifth, due to the structure of the questionnaire, it is not possible to identify the reason for treatment failure (ie, efficacy vs tolerability). In addition, treatment failure evaluated in the study encompassed all types of migraine-preventive medications that were currently available in each country at the time the survey was administered (eg, traditional oral medications, onabotulinumtoxinA for patients with CM, calcitonin gene-related peptide receptor antagonists, monoclonal antibodies, and oral gepants). Due to the structure of the survey, it is not possible to specify which therapy was not successful for the patients. Sixth, data are not available to determine whether acute therapy was successful. Seventh, the sample size was limited among the more severe categories of patients, such as those with CM. All patients had ≥ 4 MHDs, which is a low threshold of headache frequency, so the patient population could not be analyzed by subcategories of MHDs. Lastly, the SF-12 and EQ-5D-5L used in the NHWS are generic questionnaires that address general health. Therefore, the lack of differences observed across the subgroups may be due to the lack of sensitivity rather than that which may be observed using a migraine-specific questionnaire. Although there may be lack of difference using generic health instruments (ie, SF-12, EQ-5D, etc.), these tools help to conduct comparison of health status across multiple diseases and may be useful for economic evaluations. They are able to provide measures in a real-life setting that are not reflective of the measures observed in the context of clinical trials.

## Conclusions

Overall, this study demonstrated the significant burden, HCRU, indirect costs, impaired HRQoL, missed migraine-specific work and home activities days, and functional impairment among individuals with migraine, and particularly those with more preventive treatment failures. HRQoL improves when patients experience fewer headache days with successful prophylaxis. Thus, preventive treatment should be started early in the disease process to prevent decreased quality of life and reduce the burden of migraine, and there is a significant need for access to effective and tolerable migraine preventive treatment in Europe.

### Supplementary Information

**Additional file 1: Supplemental Table 1. **WPAI Questions.** Supplemental Table 2. **Baseline Characteristics Among All Participants Who Self-reported Migraine Across European Countries and the United Kingdom

## Data Availability

The dataset supporting the conclusions of this article was obtained from the 2020 National Health and Wellness Survey. The relevant data for these analyses are included in the article and its supplementary information files.

## Abbreviations

AMPP: American Migraine Prevalence and Prevention
BECOME: Burden of migrainE in specialist headache Centers treating patients with prOphylactic treatMent failure
CaMEO: Chronic Migraine Epidemiology and Outcomes
CCI: Charlson Comorbidity Index
*DSM-IV*: *Diagnostic and Statistical Manual of Mental Disorders, fourth edition*
EQ-5D: EuroQol 5-Dimension Health Questionnaire
GAD-7: Generalized Anxiety Disorder scale
GLM: Generalized linear model
GP: General practitioner
HCP: Health care provider
HCRU: Health care resource utilization
HRQoL: Health-related quality of life
MAR: Medications Adherence Reasons scale
MCS: Mental component summary
MHD: Monthly headache day
MIDAS: Migraine Disability Assessment scale
NHWS: National Health and Wellness Survey
OTC: Over-the-counter
PCS: Physical component summary
PHQ-9: Patient Health Questionnaire
SD: Standard deviation
SF-12: 12-Item Short Form Survey Instrument
UK: United Kingdom
VAS: Visual analog scale
WPAI-GH: Work Productivity and Activity Impairment General Health
