# Supplementary material for: Impact of headache frequency and preventive medication failure on quality of life, functioning, and costs among individuals with migraine across several European countries: need for effective preventive treatment
Source: J Headache Pain. 2023 Aug 24;24(1):115. doi: 10.1186/s10194-023-01655-5 (PMC10464178; doi:10.1186/s10194-023-01655-5)
Supplement: Supplementary file 1 — Additional file 1: Supplemental Table 1. WPAI Questions. Supplemental Table 2. Baseline Characteristics Among All Participants Who Self-reported Migraine Across European Countries and the United Kingdom [file 10194_2023_1655_MOESM1_ESM.docx]

**Supplemental Table 1.** WPAI Questions

| General health questions | Are you currently employed (working for pay)? *Yes, No* |
| --- | --- |
|  | During the past 7 days, how many hours did you miss from work because of your health problems? *Include hours you missed on sick days, times you went in late, left early, etc. because of your health problems. Do not include time you missed to participate in this study.*  ___ hours |
|  | During the past 7 days, how many hours did you miss from work because of any other reason, such as vacation, holidays, time off to participate in this study?  ___hours |
|  | During the past 7 days, how many hours did you actually work?  ___hours |
|  | During the past 7 days, how much did health problems affect your productivity while you were working? *Think about days you were limited in the amount or kind of work you could do, days you accomplished less than you would like, or days you could not do your work as carefully as usual. If health problems affected your work only a little, choose a low number. Choose a high number if health problems affected your work a great deal.*  <<circle a number on a line from 0 (Health problems had no effect on my work) to 10 (Health problems completely prevented me from working)>> |
|  | During the past 7 days, how much did health problems affect your ability to do your regular daily activities, other than work at a job?  *By regular activities, we mean the usual activities you do, such as work around the house, shopping, childcare, exercising, studying, etc. Think about the times you were limited in the amount or kind of activities you could do and times you accomplished less than you would like. If health problems affected your activities only a little, choose a low number. Choose a high number if health problems affected your activities a great deal.*  <<circle a number on a line from 0 (Health problems had no effect on my daily activities) to 10 (Health problems completely prevented me from doing my daily activities)>> |
| Custom items | In the past 6 months, how many days have you missed work because of your migraines? If you have not missed any days of work, please enter “0” in the space below. If you are unsure, please provide your best estimate  ___ days |
|  | In the past 6 months, how many days of household activities have you missed because of your migraines? If you are unsure, please provide your best estimate.  ___ days |

WPAI, Work Productivity and Impairment.

**Supplemental Table 2.** Baseline Characteristics Among All Participants Who Self-reported Migraine Across European Countries and the United Kingdom

|  | **European Countries + UK**  **(n=7311)** | **France**  **(n=1891)** | **Germany**  **(n=1502)** | **UK**  **(n=1611)** | **Italy**  **(n=1287)** | **Spain**  **(n=1020)** |
| --- | --- | --- | --- | --- | --- | --- |
| **Age, mean (SD)** | 43.1 (14.9) | 42.9 (15.4) | 42.7 (15.1) | 43.0 (15.0) | 45.4 (14.7) | 41.6 (13.1) |
| **Females, n (%)** | 5225 (71) | 1349 (71) | 1114 (74) | 1211 (75) | 873 (68) | 678 (66) |
| **Mean Charlson Comorbidity Index score (SD)** | 0.30 (0.91) | 0.21 (0.83) | 0.40 (0.94) | 0.29 (0.92) | 0.29 (1.02) | 0.35 (0.86) |
| **Married/living with partner, n (%)** | 4507 (62) | 1166 (62) | 771 (51) | 958 (59) | 902 (70) | 710 (70) |
| **University degree or higher, n (%)** | 3264 (45) | 948 (50) | 367 (24) | 877 (54) | 444 (34) | 628 (62) |
| **Household income, n (%)** |  |  |  |  |  |  |
| *<€20,000* | 2046 (28) | 561 (30) | 352 (23) | 500 (31) | 317 (25) | 316 (31) |
| *€20,000 to <€40,000* | 2655 (36) | 770 (41) | 442 (29) | 535 (33) | 493 (38) | 415 (41) |
| *€40,000+* | 2067 (28) | 430 (23) | 599 (40) | 450 (28) | 356 (28) | 232 (23) |
| **Labor force participation, n (%)** | 5003 (68) | 1287 (68) | 1002 (67) | 1032 (64) | 888 (69) | 794 (78) |
| **Public insurance, n (%)** | 5181 (71) | 1371 (73) | 1094 (73) | 1098 (68) | 873 (68) | 745 (73) |
| **BMI category,^a^ n (%)** |  |  |  |  |  |  |
| *Underweight: <18.5* | 353 (5) | 113 (6) | 54 (4) | 61 (4) | 77 (6) | 48 (5) |
| *Normal: 18.5 to 24.9* | 3306 (48) | 962 (52) | 636 (44) | 519 (38) | 698 (55) | 491 (49) |
| *Overweight: 25.0 to 29.9* | 2003 (29) | 517 (28) | 433 (30) | 392 (28) | 361 (29) | 300 (30) |
| *Obese: ≥30* | 1280 (18) | 264 (14) | 328 (23) | 408 (30) | 126 (10) | 154 (16) |
| **Current/former smoker, n (%)** | 4283 (59) | 1097 (58) | 835 (56) | 842 (52) | 810 (63) | 699 (69) |
| **Current alcohol use, n (%)** | 5323 (73) | 1356 (72) | 1054 (70) | 1179 (73) | 969 (75) | 765 (75) |
| **Current exercise, n (%)** | 4724 (65) | 1253 (66) | 954 (64) | 921 (57) | 838 (65) | 758 (74) |

^a^ Some participants had missing BMI data.

BMI, body mass index; SD, standard deviation; UK, United Kingdom.
